# *In Vitro* Effects of PDGF Isoforms (AA, BB, AB and CC) on Migration and Proliferation of SaOS-2 Osteoblasts and on Migration of Human Osteoblasts

**Published:** 2009-12

**Authors:** Alessandra Colciago, Fabio Celotti, Lavinia Casati, Rinaldo Giancola, Stefano M. Castano, Guido Antonini, Maria Cristina Sacchi, Paola Negri-Cesi

**Affiliations:** 1 *Department of Endocrinology, Pathophysiology and Applied Biology, University of Milano, Via Balzaretti 9, 20133 Milano, Italy*; 2 *Operative Unit of Orthopepedics and Traumatology, “S. Carlo Borromeo Hospital”, Milano, Italy*; 3 *Laboratory of Hematology, Department of Transfusional Medicine and Hematology, “SS. Antonio e Biagio e C. Arrigo Hospital” Alessandria, Italy*

**Keywords:** migration, osteoblasts, PDGF isoforms;, PRP, SaOS-2-cells

## Abstract

PDGF is a major constituent of platelet rich plasma (PRP), responsible of chemotactic and possibly of mitogenic effects of PRP on osteoblasts. PDGF family includes 5 isoforms: PDGF-AA, PDGF-AB, PDGF-BB, PDGF-CC and PDGF-DD, all expressed in platelets except PDGF-DD. Aim of this study was to analyze the effect of recombinant hPDGF-A, -AB, -B and -C, on migration and proliferation of a human osteoblastic cell line, SaOS-2. Preliminary observations on cell migration were also done in primary cultures of human osteoblasts. *In vitro* microchemotaxis and ^3^H-thymidine mitogenic assays were used. While PDGF-AB is active at concentrations present in PRP, PDGF-AA and BB are chemotactic only at much higher doses. PDGF-C is totally inactive alone or together with the active isoforms. PDGF-AA, PDGF-BB and PDGF-C stimulate SaOS-2 proliferation only at the highest dose tested, while PDGF-AB is ineffective. Primary osteoblasts are less sensitive than SaOS-2 and progressively lose responsiveness with increasing passages in culture, in line with loss of cell differentiation. The different PDGF isoforms act differentially on osteoblasts, the-AB isoform appearing the major responsible of the PRP chemiotaxis. PDGF, at the concentrations present in PRP, does not affect cell proliferation.

## INTRODUCTION

In a previous study from this laboratory (1), we have shown that a platelet rich plasma supernatant (PRP) dose-dependently stimulates both chemotaxis and cell proliferation of SaOS-2 osteoblasts, a human osteosarcoma-derived cell line. Platelet-derived growth factor (PDGF) contained in PRP appears to be the major responsible of cell migration since immunoneutralization of this growth factor almost completely inhibits chemotaxis but does not affect cell proliferation. As is well known, PDGF is one of the most abundant growth factors contained in the platelet alpha-granules and released upon platelet activation (2–4).

The PDGF family of growth factors consists of four different polypeptide chains encoded by four different genes: the classical PDGF-A and PDGF-B chains, and the more recently discovered PDGF-C and PDGF-D (5, 6). The four PDGF chains assemble into disulphide-bonded dimers via homo- or heterodimerization, and five different dimeric isoforms have been described so far: PDGF-AA, PDGFAB, PDGF-BB, PDGF-CC and PDGF-DD.

Real-time polymerase chain reaction (PCR) performed on platelet RNA revealed an apparently similar high level of expression for PDGF-A, PDGF-B, and PDGF-C (Ct values in Taqman assay 23.7, 28.1, and 27.9 respectively) but no expression was observed for PDGF-D. Likewise PDGF-A and -B, PDGF-C protein was shown by immuno-gold electron microscopy to be stored in platelet alpha granules while D chains are undetectable (5). PDGF-AA, PDGF-AB, PDGF-BB are released upon platelet activation and have been dosed in ng/mL concentration in PRPs by several authors (2, 3, 7). Western blot analysis of supernatants collected before and after platelet activation with a specific PDGF-C antibody indicate that C peptide is released by granules. Two distinct PDGF receptors, alpha and beta, mediate the effects of the PDGFs on target cells. The PDGF-A and -C chains selectively bind the alpha receptor, whereas PDGF-D preferentially binds the beta receptor and PDGF-B displays a similar affinity for both receptors. Receptor activation requires PDGF-induced receptor dimerization, leading to transphosphorylation on tyrosine. PDGF AA induces only alpha/alpha receptor dimers, PDGF AB induces alpha/alpha and alpha/beta dimers, and PDGF BB induces all 3 combinations. Although PDGF-C binds only to the alpha receptor it can produce alpha-beta heterodimer formation by transactivation of beta receptor (5).

Since in our previous studies (1) the major responsible of cell migration of PRP was PDGF, and the anti-PDGF utilized in immunoneutralization recognized both A and B peptides (the cross reactivity with the C peptide is unknown), aim of this study is to analyze the chemotactic and mitogenic effect of the different PDGF isoforms, including the PDGF-C, on SaOS-2 cells. This cell line shows a relatively well differentiated osteoblastic phenotype (8) and expresses both alfa and beta PDGF receptor (1).

SaOS-2 cells are only one model of osteoblast function and behaviour. Other osteoblast cells (9), expecially those derived by primary cultures of human osteoblasts might be of help in studying bone fracture healing. To this purpose some very preliminary studies on migration of normal human osteoblasts grown in primary cultures are also reported.

## MATERIALS AND METHODS

### Cell Culture

SaOS-2 is a mature osteoblastic cell line (American Type Culture Collection, Rockville, MD, USA) derived from a human osteosarcoma, routinely grown in DMEM supplemented with 10% fetal calf serum (FCS), glutamine (2 mM), penicillin (100 IU/mL), streptomycin (100 microg/mL) and sodium-pyruvate (1 mM).

### Platelet rich plasma preparation and activation

PRP was obtained from blood of normal healthy volunteers and prepared as previously described (1). Briefly, the platelet pellet obtained after centrifugation (platelet enrichment of about 4–5 times, 1.0–1.2 × 10^6^ platelets/microL on average) was activated with calcium gluconate/batroxobine (Pentapharm, Basel). The platelet gel was centrifuged (1400 g, 10 min, RT) and the liquid phase, enriched in growth factors (PRP in text and figures), frozen at −80°C in aliquots.

### PDGF-AA, -AB and -BB quantification

The amounts of PDGF-AA, -BB and -AB present in PRP were quantified using the quantitative sandwich enzyme immunoassay technique (Quantikine PDGF-AA, PDGF-AB, PDGF-BB Immunoassay, R&D System, Minneapolis, USA).

### Primary cultures of human osteoblasts

Human primary osteoblasts were isolated from proximal or distal epiphysis of humerus, tibia and femur of male and female patients obtained during surgical intervention for reduction of fractures. The ethics committee of the S. Carlo Borromeo Hospital, Milano, has approved the use of human specimens for this sperimentation. Trabecular bone was dissected into tiny fragments, washed with PBS and incubated in DMEM supplemented with 50% fetal bovine serum (FCS), glutamine (2 mM), penicillin (100 IU/mL), streptomycin (100 microg/mL), amphotericin B (250 microg/mL) and sodium-pyruvate (1 mM). Medium, supplemented with 20% FCS, was changed biweekly. The osteoblast phenotype was evaluated by immunolabelling with anti-osteopontin antibody (10): cells, fixed in 0.5% (p/v) paraformaldehyde +1.5% (v/v) glutaraldehyde, were incubated with a polyclonal primary antibody against osteopontin (1:200, Santa Cruz Biotechnology, USA, sc-10591) and an anti-rabbit FITC (fluorescein) conjugated secondary antibody (1:200, Amersham, USA). Cells were then observed and photographed under a fluorescent microscope. For our experiments, cells from second- to fifth passage in culture were used.

### Microchemotaxis assay

The microchemotaxis assay was performed using a 48-well Boyden's chamber according to manufacture’s instructions (Neuroprobe, Cabin John, MD). Cells were plated 48 hours before at a density of 20000 cells/cm^2^ in order to avoid confluence. For chemotaxis experiments, 28 microl of control media (DMEM) or different chemoattractants (see below) were placed in the lower compartment of the chamber, while the open-bottom wells of the upper compartment was filled with cells, collected by trypsin and resuspended in DMEM +0.1% BSA (10^5^ cells/well). Each pair of wells are separated by a polyvinylpyrrolidone-free polycarbonate porous membrane (8 micron pores) precoated with gelatine (0.2 mg/mL in PBS) for 5 days at 4°C. After migration (overnight, 37°C), cells, adherent to the underside of the membrane, were fixed by methanol and stained according to the Diff-Quik kit (Biomap, Milano, Italy). For quantitative analysis, cells were observed and counted using a 40× objective on an optical microscope. Three random objective fields of stained cells were counted for each well and the mean number of migrating cells was calculated.

### Preparation of chemoattrattants/doses utilized

The stock preparation of PRP was used at the dilution of 1:1000 in serum-free DMEM.

The final concentrations of each recombinant human PDGF isoform used in the experiment with SaOS-2 cells were: PDGF-AA and -BB (R&D System, Minneapolis, USA) 0.01 and 1 ng/mL; PDGF-AB (Peprotech, London, UK) 0.1 and 10 ng/mL; PDGF-CC (R&D System, Minneapolis, USA) 0.1, 0.5, 5, 10, 50 and 500 ng/ml. Serum-free DMEM and DMEM + 1% FCS (+10% FCS for primary osteoblasts) were added as negative and positive control respectively. These doses have been selected in order to have one dose in the range of the concentrations of the PDGF isoform present in PRP 1:1000 used in all the experiments, and one dose 100 times higher. A wider range of doses has been utilized in the case of PDGF-C since no data are available on the actual concentrations of this peptide in PRP. In the preliminary studies with primary osteoblasts PRP dilution was 1:100 and PDGF-isoforms were used at 1 and 5 ng/mL.

### Mitogenic assay

SaOS-2 cells proliferation was evaluated by [methyl-^3^H]-thymidine (Amersham Pharmacia Biotech, Buckinghamshire, England) incorporation into the DNA. Briefly, SaOS-2 cells were seeded at the density of 8000 cells/cm^2^ in 35 mm Petri dishes and grown for 24 hours in DMEM plus 10% FCS. This medium was changed to serum free-medium supplemented with different concentrations of each recombinant protein (PDGF-AA and -BB: 0.01 and 1 ng/mL; PDGF-AB: 0.1 and 10 ng/ml; PDGF-CC: 0.1, 0.5, 5, 10, 50 and 500 ng/ml) for 24 h. Cells were pulse-labeled with ^3^H-thymidine (1μCi/Petri dish) for the last 3 h of culture (37°C, 5% CO_2_); after this incubation period, cells were washed extensively with PBS without Ca^++^ and Mg^++^, incubated with 5% Trichloroacetic acid for 30 minutes at 4°C, washed with Ethanol 70% and then collected with NaOH 0.2 M. The amount of ^3^H-thymidine incorporated in the SaOS-2 cells was measured with a liquid scintillation counter (Perkin Elmer). Cells treated with DMEM and with DMEM +10% FCS were added as negative and positive control respectively.

### Real-Time PCR

Total RNA for the evaluation of the expression of alpha and beta PDGF receptors was isolated by a guanidinium isothiocyanate/phenol-chloroform extraction; 2 ug of total RNA were amplified with Real-Time PCR, using the SYBR Green method. Briefly, RNA was reverse transcribed using the High-Capacity cDNA Archive Kit (Applera Italia, Italy); Real-Time PCR was run using the SYBR Green PCR Master Mix (Applera Italia, Italy). Primers specific for selected genes were designed via the primer Express software; the efficiency of each set of primers was near 100% for both target and reference gene (GADPH). The relative quantification was evaluated by comparative CT method.

### Statistical analysis

Statistical analysis of the data was performed by one-way analysis of variance (ANOVA) followed by the Tuckey post-hoc test for multiple comparisons using the Systat Statistical software (version 5.2 for Macintosh, Evanston, IL).

## RESULTS

**Figure 1:**
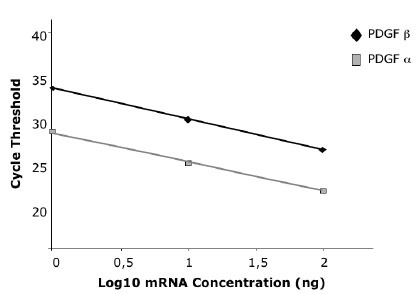
Expression of PDGF receptor alpha and beta in SaOS-2 cells. Data are the Ct values of each standard point. The line represents the regression curve.

The concentrations of the major growth factors present in our PRP preparation are shown in table 1. On the basis of PDGF-AA, -AB, -BB concentrations, we have selected the range of doses of synthetic peptides utilized in the migration and proliferation experiments, as indicated in the material and methods section. PRP also contains high concentrations of TGF-beta1 and of IGF-I, while TGFbeta2 is present in lower amounts. PDGF-CC has not been evaluated since a suitable assay is not available. The expression of the two PDGFR (alpha and beta) is represented in Figure 1. As evidenced by the Ct values, the two receptors are differentially present in SaOS-2 cells: alpha receptor has a higher expression level than beta.

**Table 1:** Mean ± SD of growth factor content in platelet rich plasma activated by a calcium/batroxobin blend

	ng/mL

PDGF-AA	7.70 ± 0.87
PDGF-AB	46.28 ± 1.39
PDGF-BB	12.14 ± 4.83
TGF-beta1	78.45 ± 1.46
TGF-beta2	0.165 ± 0.013
IGF-1	83.67 ± 9.13

**Figure 2:**
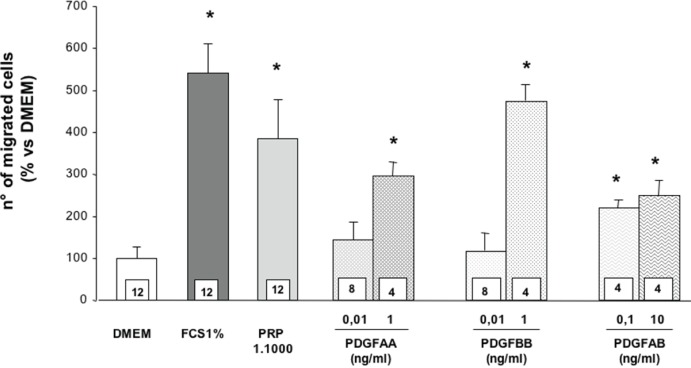
Effect of different PDGF isoforms on SaOS-2 cells migration. Values are means ± SD of the rate of migrated cells compared to DMEM. The number of samples analyzed is shown in the squares at the bottom of the columns. *p<0.05 vs. DMEM.

**Figure 3:**
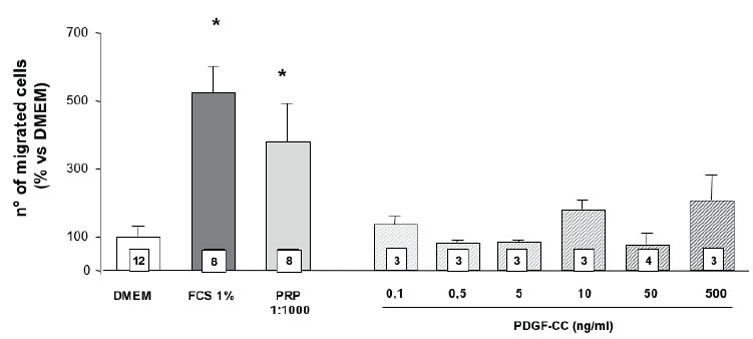
Effect of different PDGF-C doses on SaOS-2 cells migration. Values are means ± SD of the rate of migrated cells compared to DMEM. The number of samples analyzed is shown in the squares at the bottom of the columns.

The effect of the PDGF-AA, -AB, -BB isoforms on the migration of SaOS-2 osteoblasts is shown in figure 2. It appears that PRP 1:1000 is almost as effective as FCS 1%, used as positive control, and 3–4 times more effective then DMEM, the negative control. All different PDGF isoforms tested in these experiments appear active in inducing cell migration and their effect is comparable to that observed with FCS. Also, the figure 2 shows a significant different dose-response pattern, with PDGF-AA and -BB that significantly enhance chemotaxis only at the higher dose tested; moreover, at this dose, PDGF-BB has a higher effect than PDGF-AA. On the other side, PDGF-AB is moderately and equally effective at both doses tested, although the effect is reduced when compared to both PDGF-AA and -BB. Figure 3 shows the results of cell migration in presence of PDGF-C. No significant effect of this isoform is present at any dose tested (from 0.1 to 500 ng/ml). In search of possible agonist interactions between PDGF-C and the other isoforms we have analyzed the chemotactic response to both doses of PDGF-AA, -BB and -AB in presence of PDGF-C at the dose of 10 ng/ml. The results achieved show that PDGF-C does not affect the chemotactic response to the other isoforms (data not shown).

**Figure 4:**
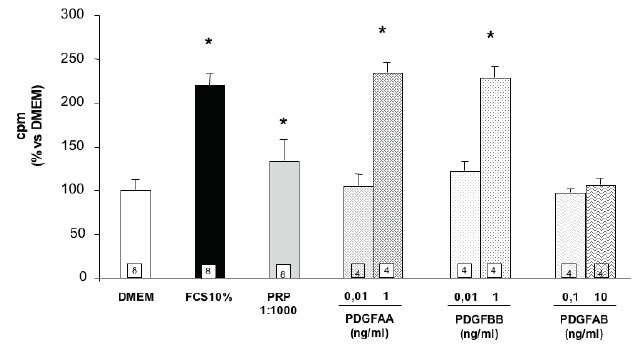
Effect of different PDGF isoforms on SaOS-2 cells proliferation. Values are means ± SD of the rate of [^3^H]-thymidine incorporation compared to DMEM. The number of samples analyzed is shown in the squares at the bottom of the columns. *p<0.05 vs. DMEM.

**Figure 5:**
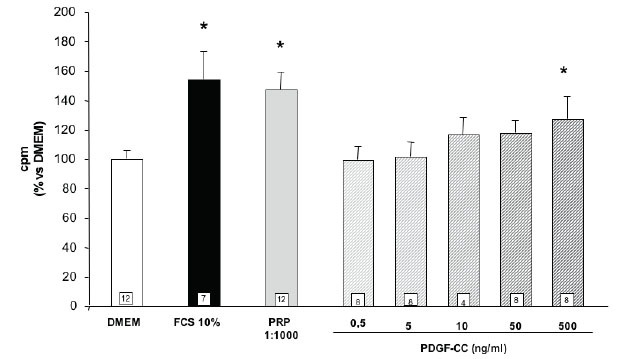
Effect of different PDGF-C doses on SaOS-2 cells proliferation. Values are means ± SD of the rate of [^3^H]-thymidine incorporation compared to DMEM. The number of samples analyzed is shown in the squares at the bottom of the columns. *p<0.05 vs. DMEM.

The effect of the different PDGF isoforms was also tested on proliferation of SaOS-2 cells. PRP 1:1000 produces a small but statistically significant stimulation of thymidine incorporation (figure 4). No effect of the lower dose of PDGF-AA, BB and AB was observed in these studies, however the higher doses of PDGF-AA and -BB, but not that of -AB, were able to increase thymidine incorporation to a level comparable with that observed in FCS treated cells (figure 4). The PDGF-C isoform stimulates cell proliferation only at the very high concentrations of 500 ng/mL (figure 5). Moreover, as in the case of the chemotactic effect, PDGF-C, added at the dose of 10 ng/mL, does not affect the proliferative response of the other isoforms (data not shown). Human osteoblasts grown in primary cultures prepared from bone specimens drawn during surgery were utilized in order to have a preliminary evaluation of their responsiveness to the chemotactic stimulus of PRP or of the biosynthetic PDGF isoforms utilized in the SaOS-2 cell studies. The cells utilized in the different experiments were isolated from 3 different patients according to the following scheme (Table 2).

**Table 2:** 

Patient sex	Age	Bone specimen from:	Study performed

Female	91	Femur	Sensitivity, Different passages
Male	34	Tibia	Morphology
Female	33	Humerus	PGDF effects

**Figure 6:**
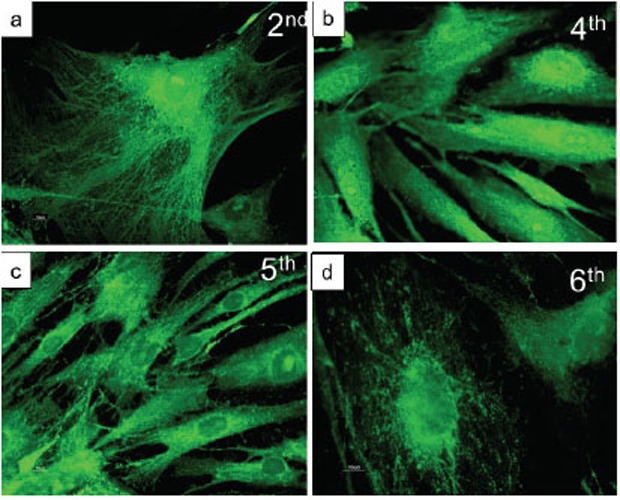
Photomicrographies of primary cultures of human osteoblasts, labelled with osteopontin (OPN). *Panels a and b*: from 2nd to 4th passage in culture the presence of OPN, a phenotypic marker for osteoblasts, is indicative of a good differentiation of these cells as osteoblasts. *Panels c and d:* from the 5th passage *in vitro*, the OPN positive cells show a progressive loss of differentiation. *Panels a and d:* original magnification 40×; *panels b and c:* original magnification 20×.

**Figure 7:**
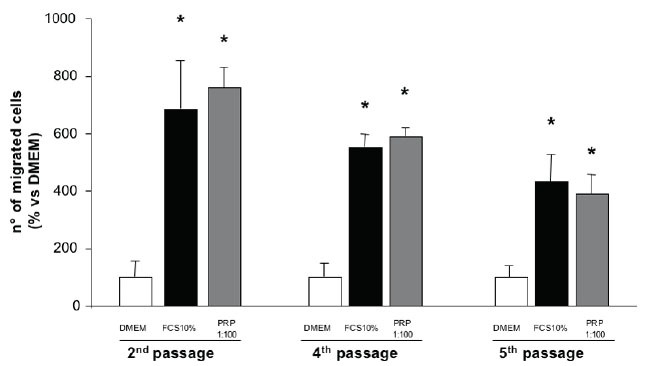
Sensitivity of primary human osteoblasts to the chemotactic effect of PRP after different passages in culture. Values are means ± SD of the rate of migrated cells compared to DMEM.

**Figure 8:**
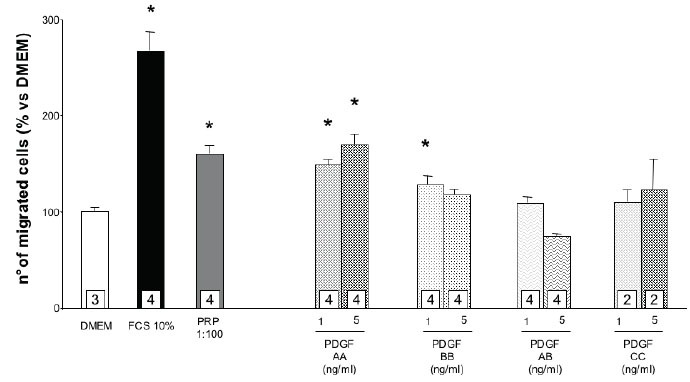
Effect of different PDGF isoforms on primary human osteoblast migration. Values are means ± SD of the rate of migrated cells compared to DMEM. The number of samples analyzed is shown in the squares at the bottom of the columns. *p<0.05 vs. DMEM

The morphological evaluation of osteoblasts by immunofluorecence for osteopontin, a marker of osteoblastic differentiation (figure 6), reveals that the primary cultures, from passage 2 to 6, are a highly homogeneous preparation of well-differentiated cells expressing the evaluated marker; from the 5^th^ passage in culture, a progressive suffering (indicated by a decreased cell dimension) seems to be present. Figure 7 shows the chemotactic response to FCS and PRP of the cultured human osteoblasts at the different passages in culture. Since in preliminary studies (data not shown) primary osteoblasts appeared less sensitive to the chemotactic stimuli than SaOS-2 cells, FCS and PRP were used at concentration of 10% and 1%. The highest migration rate occurs when the cells are at the second passage in culture while there is a progressive loss of chemotactic responsiveness in the further passages. As a consequence, cells at the second passage were used in further experiments. PRP, used at a 10% concentration, produces a significant increase of the osteoblast migration and this effect is mimicked by the PDGF-AA administration at both doses evaluated and, to a minor extent, by PDGF-BB (only at the lower dose); PDGF-AB and -CC appear ineffective (Figure 8).

## DISCUSSION

Platelet rich plasma is increasingly and effectively used in clinical practice to improve bone fracture healing due to its high content of growth factors, in particular the different PDGF isoforms, and TGF-beta1 (3).

The levels of growth factors evaluated in our platelet rich preparation containing about 1.0–1.2 × 10^6^ platelets/microL, is in the range of values, although rather variable, reported in the literature (2, 3, 11–14). In this paper we have evaluated the effect on migration and proliferation of the different PDGF isoforms, contained in the platelet granules and secreted during platelet activation, utilizing SaOS-2, a human osteosarcoma-derived cell line.

Primary cultures of normal human osteoblasts have been morphologically characterized and used in preliminar experiments, in order to find a more physiological model to study bone healing. The results obtained indicate that PDGF AA, BB and AB produce chemotactic effects on SaOS-2 osteoblasts while the C-isoform is totally inactive. Moreover, PDGF-C does not show any agonistic effect with the other active isoforms. Primary osteoblasts respond to chemotactic stimuli, even if less effectively than SaOS-2 cells, and in the study conducted on osteblasts from a single patient, PDGF AA (at both doses utilized) and BB (only at the lower dose utilized) significantly stimulate cell migration. These results are in substantial agreement with our previously published observations (1) indicating an important role of PGDF in mediating the chemotactic stimulus of PRP on SaOS-2 osteoblasts. In these studies the migratory stimulus provided by PRP was completely abolished by an antiserum able to bind both to the A and B chains of PDGF. The new data confirm that PDGF-AA, -BB and -AB are all able to produce chemotaxis: however, it must be underlined that the effective doses of PDGF-AA and PDGF-BB appear to be much higher than the actual concentration present in PRP (Table 1 and Figure 2). On the contrary, the AB isoform starts to be active at the concentration present in PRP, which is 4–6 times higher than that of the AA and BB isoform. It is therefore possible that both alpha and beta receptors should be activated at lower concentrations while a single receptor activation is sufficient at higher doses. It is noteworthy that PDGF-C, even if it binds to alpha receptors and it has been used in high concentrations, up to 500 ng/ml, is totally ineffective in inducing SaOS-2 osteoblasts chemotaxis. The lack of PDGF-CC effect might be related to the peculiar mechanism of receptor activation induced by this isoform: the relative lower expression of beta receptor in SaOS-2 cells, here demonstrated, might compromise its transactivation induced by receptor alpha binding. To the authors’ knowledge no previous report on the effects of PDGF-C on osteoblast migration is available in the literature.

In substantial agreement with our observations, Fiedler *et al*. (15) evaluated the role of PDGF in the chemoattraction of human bone-derived osteoblast at various stages of differentiation showing that rhPDGF-BB produced a powerful chemotactic stimulus, which declined with osteogenic differentiation. Moreover, the same authors (15) showed that blocking PDGF-alpha receptors with specific antibodies, the chemotactic response decreased significantly in most cases, but not to a complete inhibition of migration, whereas the preincubation with specific antibodies against the PDGF-beta receptor led to a complete inhibition of migration with all PDGF isoforms. These results are therefore indicative of the involvement of both receptor types in mediating the chemotactic stimulus that occurs in physiological situations.

It is known that cell migration is strictly related to cytoscheletal remodelling, therefore microfilaments might represent a target of PDGF action. In this contest a recent paper (16) demonstrated that the exposure of gingival fibroblasts to PRP stimulated the development of actin-enriched cellular extensions and the formation of focal adhesions. Recent data obtained in the authors’ lab suggest that a pretreatment of SaOS-2 osteoblasts with PRP might produce a PDGF induced rearrangement of the cytoscheleton related to the organization of actin filaments into lamellipodia (manuscript in preparation).

In the present study PRP 1:1000, PDGF-AA and PDGF-BB at the dose of 1 ng/mL and PDGF-C at the very high dose of 500 ng/mL stimulate SaOS-2 osteoblast proliferation, while PDGF-AB is totally ineffective. It should be underlined that the effective concentrations able to produce mitogenic effects are several fold higher than those present in PRP and that the more represented isoform, PDGF-AB, does not produce any stimulation. These results are in agreement with the observation obtained in a previous study from our laboratory (1) in which the antibody neutralization of PDGF has not produced any change in the proliferative effect of PRP. The results of our studies do not disprove the well-documented mitogenic effect of PDGF on osteoblast but only indicates that the amount of this growth factor in the PRP utilized in these studies does not appear sufficient to induce proliferative effects on SaOS-2 osteoblasts. As a matter of fact several studies document the role of PDGF in inducing proliferation of cultured human and rat osteoblasts [see Goodkin and Pierce for a review (17)]. To the authors’ knowledge no report on the effect of PDGF in SaOS-2 cells proliferation has been previously published in the literature; therefore, it is possible that, even if both types of PDGF receptors are expressed in these cells and involved in the chemotactic process, innate differences in available signalling molecules or in the regulation of receptor expression might reduce the sensitivity of SaOS-2 cells to proliferative stimuli (8, 18). The proliferative effect of PRP on osteoblasts might therefore be explained by the presence in PRP of other factors, for example IGF-I. In this setting, it could be hypothesized that IGF-I plays a role in the mitogenic effect observed in SaOS-2 osteoblasts. These cells express IGF-I-binding sites and IGF-I-treated cell lines display a dose-dependent enhanced proliferation and apopotosis suppression (19). In a recent paper, it has been shown that in SaOS-2 cells the addition to PRP of a neutralizing antibody against PDGF, TGF-beta1 and IGF-I, significantly suppressed proliferation compared at 36 hours; however, at 72 hours, only the neutralizing antibodies of PDGF and TGF-beta1, but not IGF-I, significantly suppressed proliferation (20). These apparently conflicting data reflect the complex interactions among the different factors involved in cellular proliferation.

The primary cultures of human osteoblasts utilized in our preliminary studies appear to be composed by a homogeneous population of cells expressing osteopontin, indicative of a definite differentiation of these cells as osteoblasts. These cells seem to progressively lose their degree of differentiation with the increasing number of passages in culture. This difference is in agreement with a loss of responsiveness to chemotactic stimuli. The data, obtained from primary cultures coming from human bone specimens, even if quantitatively limited, seem to confirm the observations obtained with SaOS-2 cells.

From the data here reported it might be concluded that the different PDGF isoforms act differentially on osteoblasts: the-AB isoform is the major responsible of the PRP chemiotaxis, while the C-isoform is totally inactive. Moreover, PDGF-C does not show any agonistic effect with the other active isoforms. PDGF, at the concentrations present in PRP, does not affect cell proliferation.
